# Malnutrition, Depression, Poor Sleep Quality, and Difficulty Falling Asleep at Night Are Associated with a Higher Risk of Cognitive Frailty in Older Adults during the COVID-19 Restrictions

**DOI:** 10.3390/nu15132849

**Published:** 2023-06-23

**Authors:** Jiranan Griffiths, Mathuramat Seesen, Wachiranun Sirikul, Penprapa Siviroj

**Affiliations:** 1 Department of Occupational Therapy, Faculty of Associated Medical Sciences, Chiang Mai University, Chiang Mai 50200, Thailand; jiranan.gr@cmu.ac.th; 2 Department of Community Medicine, Faculty of Medicine, Chiang Mai University, Chiang Mai 50200, Thailand; mathuramat.s@cmu.ac.th (M.S.); wachiranun.sir@cmu.ac.th (W.S.); 3 Center of Data Analytics and Knowledge Synthesis for Health Care, Chiang Mai University, Chiang Mai 50200, Thailand

**Keywords:** older adults, cognitive frailty, malnutrition, depression, sleep quality, falling asleep at night, COVID-19 restrictions

## Abstract

The COVID-19 restrictions, such as social isolation and disruption of daily routines, can have detrimental effects, including increased stress, anxiety, sleep disturbance, and physical and cognitive decline among older adults. This study aimed to examine the association between nutritional status, depression, sleep quality, falling asleep at night, and cognitive frailty (CF) among older Thai adults during the COVID-19 pandemic. This cross-sectional study included 408 older adults with an average age of 70.54 (5.49) years. CF was determined using Fried’s frailty phenotype and the Montreal Cognitive Assessment Basic. The Mini Nutritional Assessment-Short Form, Pittsburgh Sleep Quality Index, and geriatric depression assessment were used for assessment. Multiple logistic regression analysis demonstrated that participants who were malnourished (OR 3.786; 95%CI 1.719–8.335), depressed (OR 5.003; 95%CI 2.399–10.434), had poor sleep quality (OR 1.613; 95%CI 1.041–2.500), and engaged in difficulty falling asleep (OR 1.809; 95%CI 1.022–3.203) had a higher risk of CF compared to those who did not exhibit these factors. Therefore, malnutrition, depression, poor sleep quality, and difficulty falling asleep were identified as risk factors for CF among older adults in Thailand linked to the impact of the COVID-19 pandemic. It is crucial to develop interventions to prevent CF resulting from the mentioned variables.

## 1. Introduction

The COVID-19 pandemic has had a significant impact on people’s lifestyles worldwide due to the implementation of measures such as lockdowns and social distancing, which have been enacted with the purpose of mitigating the transmission of the virus. These measures have disrupted individuals’ daily routines encompassing physical activities and sleep patterns, thereby engendering feelings of loneliness, depression, and sleep problems, particularly affecting older adults [1,2]. Furthermore, limitations imposed on outdoor activities led to a decline in physical function, manifesting as the loss of muscle mass, reduced mobility, and an increased risk of non-communicable diseases [3,4]. Moreover, a decrease in time spent on physical activities may also have adverse effects on cognitive function [5]. A systematic review with meta-analysis has revealed that cognitive decline is associated with physical frailty in older adults [6].

Cognitive frailty is a clinical condition observed in older individuals who exhibit both physical frailty and cognitive impairment but do not meet the criteria for dementia. This vulnerable state is considered a precursor to the neurodegenerative processes that eventually lead to dementia [7,8]. Cognitive frailty arises from the complex interplay of factors such as chronic inflammation, oxidative stress, and elevated factor VIII. Chronic inflammation harms neurons and disrupts intercellular communication, leading to cognitive decline. Oxidative stress occurs due to an imbalance between reactive oxygen species production and antioxidant defenses. Factor VIII is a blood clotting factor that has been linked to an increased risk of cognitive impairment and dementia. These processes can lead to neuronal damage, compromised cognitive function, and the manifestation of cognitive frailty in older adults [9,10,11].

Frailty, on the other hand, represents a distinct phenotype within a clinical syndrome characterized by a reduction in physiological reserves, an increased susceptibility to stressors, and a higher risk of adverse outcomes [12]. According to Fried’s criteria, physical frailty involves a decline in physical function, including reductions in strength, endurance, balance, walking performance, and activity levels [13]. Mild cognitive impairment (MCI), in turn, denotes a stage of cognitive decline that falls between normal age-related cognitive impairments and dementia. MCI encompasses various cognitive functions such as memory, attention, language, and executive function [14,15,16]. When physical frailty and MCI coexist, the ability of older adults to perform daily activities and maintain their independence becomes compromised. This combined condition increases the risk of adverse health outcomes, including falls, hospitalization, disability, and mortality [17,18]. To identify such impairments, the mini-mental state examination (MMSE) and the Montreal Cognitive Assessment (MoCA) are two widely recognized tests used for screening cognitive impairment. The MoCA test, in particular, has gained recognition as a sensitive and reliable method for detecting MCI, while the MMSE exam remains an accepted tool for screening generalized cognitive decline or dementia [19]. A previous systematic review and meta-analysis study reported that the prevalence of cognitive frailty among community-dwelling older adults in various countries was 16.0% [20]. Furthermore, our previous study conducted in Thailand yielded a prevalence of 28.7% [21]. Cognitive frailty is strongly associated with an elevated risk of multiple adverse health outcomes, such as malnutrition, mental health problems, disability, and mortality [18,22,23,24].

Malnutrition refers to a condition characterized by an inadequate intake of nutrients, an imbalance of vital nutrients, or an impaired ability to utilize nutrients effectively [25]. Insufficient intake of essential nutrients can give rise to various health problems, such as weakness, fatigue, and an increased risk of cognitive decline and dementia in older individuals [7,26]. Furthermore, malnutrition can lead to chronic inflammation, which in turn can cause damage to brain cells, impair cognitive function, and contribute to cognitive frailty [26,27]. However, studies on the association between malnutrition and cognitive frailty in community-dwelling elderly are limited. Previous studies have indicated that older individuals with cognitive frailty tend to have lower scores on the Mini Nutritional Assessment-Short Form (MNA-SF), suggesting a poorer nutritional or malnourished state compared to those without cognitive frailty [21,27].

Depression is a mood disorder that affects a person’s feelings, thoughts, and behavior and is characterized by symptoms such as sadness, helplessness, and loss of interest in activities [28]. Previous studies have identified a strong association between physical frailty and depression in older individuals [29,30]. Furthermore, the evidence suggests that depression contributes to the development of persistent or progressive cognitive decline [31,32]. Cognitive impairment can decrease a person’s ability to engage in activities, perception of social isolation, stress management, and negative emotions, all of which can contribute to the onset or exacerbation of depression. Consequently, depression increases the risk of developing cognitive frailty [32,33].

There is a connection between frailty, cognition, and sleep. Previous studies have demonstrated an association between sleep quality and napping duration, physical frailty, and cognitive impairment. Sleep quality holds significant importance in the overall health of older individuals, as it is an integral part of their daily lives [34,35,36,37]. Sleep quality refers to an individual’s subjective experience of the quantity and effectiveness of sleep obtained during a specific period. Poor sleep quality is typically characterized by difficulties falling asleep, frequent awakening during the night, and a constant feeling of tiredness. It is worth noting that poor sleep is a common issue among older adults [34,38]. Difficulty falling asleep at night has the potential to disrupt the natural circadian rhythm and has been associated with cognitive frailty [36,37,39,40]. Furthermore, a previous study highlighted that more than half of the participants experienced altered sleep patterns and heightened psychological distress during the COVID-19 lockdown [41].

Current studies indicate a connection between malnutrition, depression, poor sleep quality, and falling asleep at night time with cognitive frailty in older individuals. However, further research is necessary to confirm these associations under the COVID-19 restrictions. The aging population in Thailand has grown rapidly, with reports stating that in 2021, the number of old individuals reached 12.5 million out of a total population of 66.7 million (18.7% of the total population) [42]. From 3 January 2020 to 7 June 2023, Thailand recorded 4,745,043 confirmed COVID-19 cases and 34,163 fatalities [43]. To reduce the risk or reverse cognitive frailty in older adults, it is essential to prioritize nutritional status, mental health (such as depression, anxiety, and stress), maintaining good sleep hygiene, managing sleep problems, and limiting the duration of falling asleep at night time. However, there is a lack of evidence regarding cognitive frailty in older adults in relation to nutritional status, mental health problems, and sleep quality during the COVID-19 restrictions in Thailand. We conducted this study among older adults aged over 65 years residing in Chiang Mai, Thailand. The rationale behind selecting this cohort lies in their susceptibility to the infectious disease, given the initial phase of heightened risk they experienced during the onset of the COVID-19 pandemic’s first wave, spanning from June to December 2021 [43]. The aim of this study was to examine the association between nutritional status, depression, sleep quality, falling asleep at night, and cognitive frailty in community-dwelling older adults during the COVID-19 pandemic.

## 2. Materials and Methods

### 2.1. Study Design and Participants

This cross-sectional study was conducted in Khua Mung Subdistrict, Saraphi District, Chiang Mai Province, Thailand, during the period of July to August 2021, which coincided with the COVID-19 pandemic restrictions. Details regarding participant recruitment, sample calculation, sampling design, and inclusion and exclusion criteria are all described in our previously published study [21]. In this study, we provide a brief summary of those aspects.

**Figure 1:**
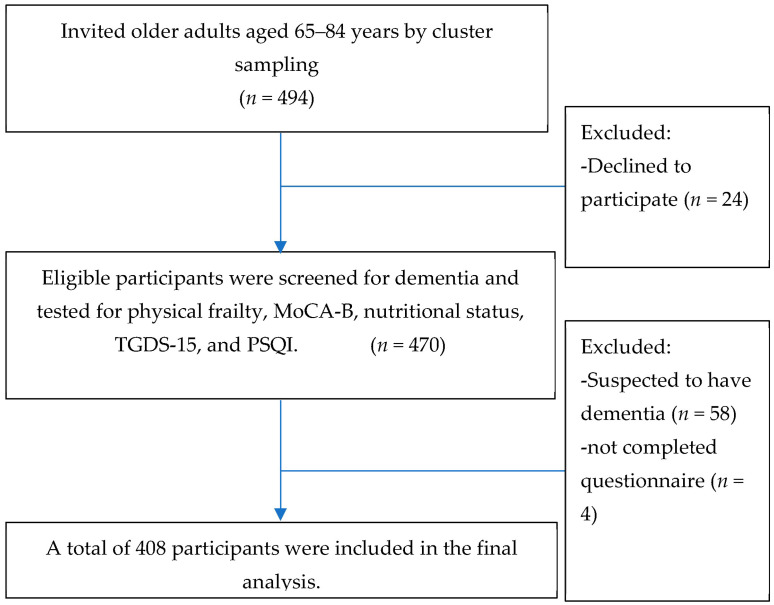
Diagram of the study participant selection. Abbreviations: MoCA-B, Montreal Cognitive Assessment-Basic; TGDS-15, Thai version of 15-item Geriatric Depression Scale; PSQI, Pittsburgh Sleep Quality Index.

To calculate the sample size, we used a population of 934 individuals, and the expected frequency was determined based on a study by Chye L. et al. [22], which reported a prevalence of 1.6% for cognitive frailty among older adults. We established a 95% confidence interval, conducted a two-sided hypothesis test with a significance level of 0.01, and aimed for a power of 90%. As a result, the final sample consisted of 494 individuals. To explore the factors associated with cognitive frailty, seven potential factors were considered: age, educational level, diabetes, nutritional status, depression, sleep quality, and falling asleep at night. According to the rule of events per variable (EPVs), a multivariable binary logistic regression requires at least 10 EPVs [44]. Therefore, at least 70 cognitively frail cases are expected from a multivariable analysis of all potential associated factors. In order to exclude those with specific conditions, we reviewed the health-promoting hospital database and excluded those who had been diagnosed with dementia, depression, end-stage kidney disease, hepatitis, cirrhosis, autoimmune disorders, cancer, acute trauma, acute illnesses, or steroid. Additionally, the Mental Status Examination Thai 10 (MSET10) was utilized to identify those with suspected dementia, which were further excluded from the research analysis, as cognitive frailty is defined as a combination of mild cognitive impairment (MCI) and frailty without evidence of dementia. In our previous study published in Nutrients [21], participants with Thai Geriatric Depression Scale (TGDS) scores exceeded 6, indicating probable depression, were excluded. Intriguingly, this study revealed that depression, as determined by TGDS scores, posed a risk factor for cognitive frailty. The participants in this study were sourced from the same population. Finally, a total of 408 participants were included in the data analysis (Figure 1).

### 2.2. Measurements

Data collection was performed by 10 medical students using a questionnaire. The questionnaire covers a range of demographic and health characteristics, including age, sex, marital status, educational level, and history of underlying diseases such as hypertension, type 2 diabetes, heart disease, dyslipidemia, and osteoporosis/gout. Additionally, it assessed alcohol and tobacco use, nutritional status using the Mini Nutritional Assessment-Short Form (MNA-SF), and sleep quality using the Pittsburg Sleep Quality Index (PSQI). To identify individuals with dementia, the Mental Status Examination Thai 10 (MSET10) was employed. MSET10 is based on the validated and modified MMSE Thai 2002 [45]. It has a total score of 29, with cutoff scores of 22 for individuals who completed primary school, 17 for those who did not, and 14 for illiterates. Furthermore, the participant’s body mass index (BMI) was measured alongside cognitive frailty. According to the Asia-Pacific regional guideline on BMI for Asian individuals, a BMI below 18.5 kg/m^2^ indicates underweight [46].

#### 2.2.1. Cognitive Frailty Assessment

In this study, cognitive frailty was defined as the coexistence of physical frailty and cognitive impairment without dementia. Both physical pre-frailty and physical frailty were considered as forms of physical frailty [7,8]. The details regarding the assessment of physical frailty and cognitive impairment are provided below:

(1) Physical frailty assessment: We employed Fried’s frailty phenotype, which is characterized by meeting three or more of the following criteria. Individuals who meet one or two criteria are classified as pre-frail [13,47]. The set of five criteria includes:
  - Unintentional weight loss: a loss of more than 4.5 kg in the past year that was not intentionally pursued.
  - Self-reported exhaustion: persistent feelings of exhaustion or weariness, even after sufficient rest.
  - Weakness: grip strength was measured using a digital hand dynamometer (TAKEI T.K.K.5401^®^, Takei Scientific Instruments Co., Ltd., Tokyo, Japan). Grip strength in the lowest 20% for their gender and body mass index (BMI).
  - Slow walking speed: the 15-foot (4.57-m) walking test was conducted with participants instructed to walk at their normal pace. Walk time is stratified based on gender and height.
  - Low physical activity: Engagement in physical activity of less than 383 kcal per week.
(2) Cognitive Function Assessment: we used the Thai version of the Montreal Cognitive Assessment Basic (MoCA-B) to evaluate cognitive function. MoCA-B is a modified version of the original MoCA specifically designed for individuals with low education levels [48,49]. Tasks that required literacy were eliminated, and literacy-independent tasks that assessed the same cognitive function were introduced. The MoCA-B has undergone validation among Thai elders in the community with low education levels and has demonstrated excellent discriminatory performance in screening for MCI. The paper-based test comprises ten cognitive domains, including executive function, immediate recall, fluency, orientation, calculation, abstraction, delayed recall, visuoperception, naming, and attention. The maximum achievement score is 30, with a cut-off score of 24 indicating MCI [48]. The training and supervision for the test were provided by a certified occupational therapist and academic who possesses certification number THGRIJI69617-02 in the Montreal Cognitive Assessment (MoCA), given by Dr. Nasreddine, Ziad.

#### 2.2.2. Mini Nutritional Status Assessment-Short Form

The Mini Nutritional Assessment-Short Form (MNA-SF) is commonly used to evaluate the nutritional status of the elderly in various settings, particularly among community-dwelling older adults [50,51,52,53]. The MNA-SF has demonstrated high sensitivity in detecting malnutrition and a strong correlation with full Mini Nutritional Assessment in the population [51]. However, it should not be solely relied upon for making a diagnosis. While the MNA-SF can be a useful tool to identify elderly individuals at risk of malnutrition or those with a malnourished status, it should be used in conjunction with other diagnostic methods. The MNA-SF comprised six questions related to dietary intake, weight loss, mobility, psychological stress or acute disease, and neuropsychological problems. Scoring is as follows: a score of 12 to 14 indicates “normal nutritional status”, a score of 8 to 11 indicates “at risk of malnutrition”, and a score of less than 8 indicates “malnourished”; 14 is the highest score [51,54,55].

#### 2.2.3. Depression Assessment

The Geriatric Depression Scale (GDS) is a self-reported questionnaire that assesses the presence and severity of depression in older adults. These questions cover various symptoms, including mood, energy, appetite, and sleep [56,57,58]. In this study, we utilized the Thai version of the 15-item Geriatric Depression Scale (TGDS-15), which has been proven to be an effective screening tool for major depressive disorder in Thai elderly individuals. The maximum score on this scale is 15, and a score greater than 6 indicates probable depression [56,59,60].

#### 2.2.4. Sleep Quality Assessment

To assess sleep quality over a one-month period, we employed the Pittsburgh Sleep Quality Index (PSQI). The self-rated questionnaire consists of seven components: subjective sleep quality, sleep latency, sleep duration, habitual sleep efficiency, sleep disturbance, use of sleeping medication, and daytime dysfunction [61]. The Thai version of the PSQI has been tested among older adults in Thailand, with translation demonstrating good inter-rater agreement (>80%) [62]. Additionally, the global scores of the Thai-PSQI exhibited excellent internal consistency, as measured by Cronbach’s alpha (0.837) [63]. Each component is scored on a scale from 0 to 3, with higher scores indicating poorer sleep quality [64,65]. The component scores are summed to yield a global score ranging from 0 to 21, where higher scores suggest worse sleep quality. A PSQI score greater than 7 indicates poor sleep quality.

#### 2.2.5. Falling Asleep at Night

To collect data on difficulty falling asleep at night, we asked participants the following question: “During the past month, how long (in minutes) has it usually taken you to fall asleep each night?” In this study, a long period of falling asleep specifically referred to the difficulty of falling asleep at night. Based on the relevant literature [66], participants were categorized into two groups: (1) individuals engaging in short periods of less than 30 min; and (2) those with longer periods lasting more than 30 min.

### 2.3. Statistical Analysis

All data were analyzed using the IBM SPSS statistical package ver. 28.0 for Windows (IBM Corp., Armonk, NY, USA). Descriptive statistics were used to present the means ± standard deviations (SD) for continuous variables and percentages for categorical variables. The normality of continuous data was assessed using the Shapiro–Wilk test. Variables that were not normally distributed were reported as median (IQR), while normally distributed variables were reported as mean (SD). The chi-square test or Fisher's exact test was employed to analyze the distribution of differences within demographics, nutritional status, sleep quality, falling asleep at night, and cognitive frailty. The comparison of median value of sleep quality components between cognitively frail and non-cognitively frail groups was performed using the Mann–Whitney U test. The association between nutritional status, depression, sleep quality status, falling asleep at night, and cognitive frailty was assessed using binary logistic regression. Additionally, multiple logistic regression with stepwise forward selection was utilized to analyze the association of significant variables identified in binary logistic regression, such as age, educational level, current drinking, history of diabetes millets, nutritional status, sleep quality, and falling asleep at night. All statistical tests were two-sided, and a *p*-value of 0.05 or lower was considered statistically significant. The study’s method and results adhered to the STROBE (strengthening the reporting of observational studies in epidemiology) recommendations for cross-sectional studies [67].

### 2.4. Ethical Considerations

All participants provided their informed consent to be included before they participated in the study. The protocol was approved by the Ethics Committee of Faculty of Medicine, Chiang Mai University (Ethical number: COM-2564-08031; Date of approval: 22 April 2021). The study was conducted in accordance with the Declaration of Helsinki.

## 3. Results

### 3.1. The Characteristics of the Participants

A total of 408 participants were included in this study, with 241 females (59.1%) and 167 males (40.9%). The average age of the participants was 70.54 years (±5.49), and 9.8% of them were aged 80 years and older. The majority of participants had low education (90.5%). Among the participants, the most prevalent conditions were hypertension (52.5%), dyslipidemia (17.6%), and diabetes mellitus (16.4%). Additionally, 15.0% of the participants reported current alcohol consumption, while 10.6% had a BMI indicating underweight. Notably, the participants with cognitive frailty were significantly older, aged ≥80 years (*p* < 0.001), had low levels of education (*p* < 0.001), were diagnosed with diabetes mellitus (*p* = 0.015), and reported no alcohol consumption (*p* = 0.003) (Table 1).

### 3.2. Cognitive Frailty, Physical Frailty, and Cognitive Impairment

**Figure 2:**
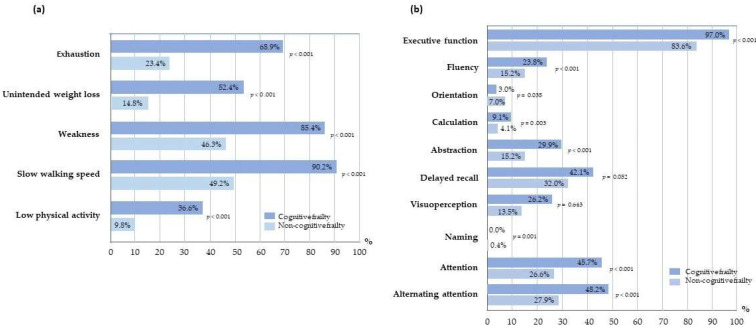
The histogram illustrates the proportion for (**a**) physical frailty and its characteristics and (**b**) mild cognitive impairment and all components in cognitively frail and non-cognitively frail groups. The differences between two groups were analyzed by Fisher’s Exact test.

The prevalence of cognitive frailty among older adults in this study was 34.8% (164 out of 470 participants). Physical frailty and cognitive impairment were prevalent in 41.9% and 84.6%, respectively. Figure 2 and Supplementary Table S1 present the differences in the proportion of physical frailty indicators and domains of cognitive function impairment between cognitively frail and non-cognitively frail groups. Participants in the cognitively frail group demonstrated a significantly higher proportion of all frailty indicators, including exhaustion, underweight loss, handgrip weakness, slow walking speed, and low physical activity, compared to the non-cognitively frail group (*p* < 0.001) (Figure 2a). Additionally, the cognitively frail group exhibited a higher proportion of impairment across all domains of cognitive function, such as executive function, fluency, orientation, calculation, abstraction, naming, attention, and alternative attention, except for delayed recall and visuoperception, when compared to the non-cognitively frail group (Figure 2b).

### 3.3. Nutritional Status, Depression, Sleep Quality, and Falling Asleep at Night

Table 2 displays the nutritional status assessed using MNA-SF, depression levels measured by TGDS-15, sleep quality evaluated through PSQI, and the occurrence of falling asleep at night, along with the difference in proportions of these parameters between cognitively frail and non-cognitively frail groups. The finding revealed that a majority of participants were either at risk of malnutrition (58.1%) or malnourished (10.3%). Additionally, 12.0% of participants experienced depression, 49.5% had poor sleep quality, and 16.9% reported engaging in difficulty falling asleep at night. There were significant associations between cognitive frailty and nutritional status (*p* ≤ 0.001), depression (*p* ≤ 0.001), sleep quality (*p* ≤ 0.001), and falling asleep at night (*p* ≤ 0.001). The cognitively frail group exhibited a higher proportion of participants classified as malnourished (17.7%), experiencing depression (23.2%), having poor sleep quality (56.7%), and having difficulty falling asleep at night (22.6%) compared to the non-cognitively frail group. Furthermore, for more detailed information, including questions and components, refer to Supplementary Tables S2–S5, which provide descriptions of MNA-SF (see Supplementary Table S2), TGDS-15 (see Supplementary Table S3), and PSQI (Supplementary Tables S4 and S5).

### 3.4. Associations between Nutritional Status, Sleep Quality, Falling Asleep at Night, and Cognitive Frailty

Binary logistic regression and multivariate logistic regression, using the forward stepwise technique, were employed to analyze the associations between nutritional status, depression, sleep quality and falling asleep at night, and cognitive frailty. The results, presented in Table 3, include both unadjusted and adjusted values accounting for potential confounders such as age, education level, a history of diabetes mellitus, and current drinking. The findings demonstrate that individuals with poor nutrition, depression, bad sleep quality, and difficulty falling asleep at night had a significantly higher prevalence of cognitive frailty compared to those in the non-cognitively frail group who had normal nutrition, were not depressed, had good sleep quality, and fell asleep easily at night (Model A). After adjustment, there was a higher risk of cognitive frailty in participants with malnourished nutrition (Adjusted odds ratio (AOR) 3.786, 95%CI 1.719 to 8.335, *p* = 0.001) (Model B). Participants with malnourished nutrition (AOR 3.499, 95%CI 1.547 to 7.914, *p* = 0.003) and depression (AOR 5.003, 95%CI 2.399 to 10.434, *p* < 0.001) had a higher risk of cognitive frailty (Model C). Similarly, malnourished nutrition (AOR 3.498, 95%CI 1.576 to 7.767, *p* = 0.002) and bad sleep quality (AOR 1.613, 95%CI 1.041 to 2.500, *p* < 0.032) were associated with a higher prevalence of cognitive frailty (Model D). In model E, a higher prevalence of CF was associated with malnourished nutrition (AOR 3.715, 95%CI 1.675 to 8.237, *p* = 0.001) and difficulty falling asleep at night (AOR 1.809, 95%CI 1.022 to 3.203, *p* < 0.042).

## 4. Discussion

This study is the first investigation to study the association between combining nutritional status, depression, sleep quality, falling asleep at night, and cognitive frailty in Thai community-dwelling older adults during the COVID-19 restrictions. The findings indicate that older adults in Thailand who experienced malnutrition, depression, poor sleep quality, and difficulty falling asleep at night had a higher risk of cognitive frailty compared to those who did not experience these factors. Our study revealed that 34.8% of older adults were affected by cognitive frailty. It is important to acknowledge that there is variation in the prevalence of cognitive frailty, which underscores the need for a cautious interpretation of prevalence results. The prevalence observed in this study was higher than that in our previous study in the same population (28.7%) [21] because we did not exclude probable depression using the TGSD score. Furthermore, our prevalence rate was higher when compared to international studies [20,22,36]. We collected data during the initial peak of the first wave of the COVID-19 pandemic in Thailand [43].

It is crucial to recognize that social isolation or restriction measures have a significant impact on both individual and family behaviors. These measures affect eating habits, engagement in exercise or physical activities, and sleep patterns [1,2]. These lifestyle factors have a role in influencing physical frailty and impaired cognitive function, which contribute to cognitive frailty. Additionally, variations in prevalence rates across studies may arise from the use of different methods to assess cognitive frailty, physical frailty, and cognitive function. These lifestyle factors have a role in influencing physical frailty and impaired cognitive function, which contribute to cognitive frailty. Another reason for a higher prevalence in our study is that we recruited participants aged 65 years and older. In contrast, previous studies included older adults over the age of 55 or 60 years [20,22]. It is important to note that the prevalence of cognitive frailty generally increases with age [68]. Moreover, our findings indicate that only 1.8% of participants with cognitive frailty had received education beyond the primary level, which is typically considered a low level of education. A previous study found a correlation between higher education among community-dwelling older adults and a slow decline in cognitive function. Interestingly, low education was identified as a risk factor for physical frailty [69,70,71]. Our results also showed that old age and low education were risk factors for cognitive frailty. Higher education has been associated with improved health literacy, which, in turn, has been related to a reduced risk of frailty [71].

Our findings showed that older adults who are malnourished are more likely to experience cognitive frailty compared with those who are not. Specifically, 10.3% of the study participants were identified as malnourished; they exhibited a heightened risk of cognitive frailty. This aligns with previous studies that have consistently identified malnutrition as a substantial risk factor for cognitive frailty. The deleterious impact of malnutrition on critical physiological factors, including muscle mass, muscle strength, and functional independence as daily activities, is well-documented [26,27]. This adverse effect can primality be attributed to inadequate protein intake, which results in diminished energy levels and compromised bodily function [7,26]. We conducted a multivariate logistic regression analysis was undertaken to investigate the influence of additional factors on the manifestation of cognitive frailty. These factors, namely depression, poor sleep quality, difficulty falling asleep at night, and malnutrition, were comprehensively considered. This examination yielded compelling findings, disclosing a heightened prevalence of cognitive frailty.

Notably, the association between depression and cognitive frailty in this study is consistent with previous studies that found that depressive symptoms contribute to the development of cognitive frailty. Specifically, individuals exhibiting slow walking and increased gait variability demonstrated a higher rate of depression, leading to decreasing physical activity and subsequent muscular weakness [32,33]. Additionally, older adults with depression showed reduced executive function and slower processing speed, resulting in instability during walking [72]. These effects can be attributed to structural changes in the brain, including lower gray matter volume, subcortical volume, and cerebral white matter volume, as well as disruptions in connectivity underlying depressive symptoms, alongside reduced muscle mass [31,73,74,75]. Moreover, our study revealed a significant association between depression, malnutrition, and cognitive frailty, consistent with a previous study [29,30]. Common pathways linking depression and malnutrition may contribute to compounded effects, such as weight loss, activation of proinflammatory cytokines, and increased oxidative stress. Additionally, depression-induced loss of appetite can lead to significant weight loss, physical frailty, and compromised nutritional status [76].

Difficulties falling asleep at night before bedtime is associated significantly with cognitive frailty. This study revealed that older adults with poor sleep quality and difficulty falling asleep were more likely to experience cognitive frailty. This is consistent with a study in China, which found that poor sleep quality and a long nap duration at nighttime were associated with a high risk of cognitive frailty in older people in nursing homes. Short nap duration was associated with a low prevalence of cognitive frailty [36]. A systematic review showed that the prevalence of sleep disturbances in older adults with MCI and Alzheimer’s disease is higher than in healthy older people [77]. Our finding observed that older adults with cognitive frailty exhibited a notably higher prevalence of poor sleep quality (56.7%) in comparison to those without cognitive frailty. Sleep deprivation is a well-documented consequence of poor sleep quality and contributes to the accumulation of deleterious substances, such as free radicals and neurotoxic waste, within the brain. This accumulation is linked to cognitive decline and is associated with poor sleep [78]. A previous meta-analysis revealed that poor sleep quality contributes to increased levels of inflammatory markers such as C-reactive protein (CRP) and factor VIII. These changes in the immune system are considered primary causes of frailty [9,10,11,79]. Furthermore, a previous study indicated that individuals with cognitive impairment had higher levels of CPR [80]. A study in a rural area in the north of Thailand showed that the prevalence of poor sleep quality among the elderly in the study area was high, and the major significant predictors of poor sleep quality were mild depression and poor family relationships [81].

Additionally, a previous study reported that sleep quality was poor and had a negative impact on mental health during the COVID-19 pandemic [41]. During the COVID-19 pandemic, older people may experience sleep difficulties due to the increase in stress and anxiety. They may be concerned about the virus and its effect on their health, especially if they have other chronic diseases. The pandemic disrupted the daily activities of many people, including older individuals. Changes in regular schedules reduced physical activities, and altered social interaction could disrupt sleep and wake cycles and may make it more difficult to fall asleep or lead to poor sleep quality [82]. Social isolation restricts social gatherings and limits contact with family and friends and can lead to loneliness and depression, which can negatively affect sleep patterns [1,2]. The data collection in our study was during the initial peak of the first wave of COVID-19, which had a higher rate of infection and death [43]. Financial concerns in their family during the COVID-19 pandemic can lead to increased stress and may make it harder for them to fall asleep [83]. Helping older people to sleep well is good for preventing cognitive frailty; we should encourage older adults to have regular physical activity at home by keeping active by, for example, performing gentle housework, walking, gardening, and watering plants [84]. Maintaining a regular sleep schedule could promote a better quality of sleep patterns. A study in Japan showed that a short daytime napping (less than 30 min) reduced the risk of cognitive decline over 5 years among older people in the community [85].

Apart from the risk factors that we mentioned above, a factor associated with cognitive frailty in our study is diabetes mellitus. A study reported that it has a significant association with cognitive frailty and hypoglycemia and is likely to play a role in the development of cognitive frailty. The relationship between physical frailty and cognitive impairment is circular [86]. Diabetes can indeed disrupt sleeping patterns; if the blood sugar level is too high, it can lead to frequent urination and discomfort, and that has an effect on sleeping [87,88]. It is important to note that a balanced and varied diet is crucial for overall health. Healthcare professionals should combine recommendations on a healthy diet, physical activity, cognitive stimulation, mental health support, and regular sleeping patterns so that it could help to prevent cognitive frailty in older adults.

The study’s notable strength lies in the exclusion of participants with a high suspicion of dementia, reducing the risk of misdiagnosing cognitive frailty. However, it is important to acknowledge several limitations. Firstly, due to the cross-sectional nature of the study design, establishing a causal relationship between variables was not feasible. Secondly, the assessment of older adults relied on self-reported questionnaires, introducing the potential for information bias. Thirdly, this study did not investigate the long-term effects of the pandemic on nutritional status and mental health issues.

## 5. Conclusions

Malnutrition, along with depression, poor sleep quality, and difficulty falling asleep at night, were all substantially linked to cognitive frailty, increasing the risk for community-dwelling older adults as a result of Thailand’s COVID-19 pandemic restrictions. To address this, it is recommended to monitor the nutritional status, mental health, and sleep quality of older adults concurrently and implement effective prevention programs that ensure adequate nutrition, reduce depression, improve sleep quality, and promote falling asleep easily at night. Further studies are needed to validate these associations between the mentioned variables and cognitive frailty in older adults, as well as to examine their long-term impacts. There is a need for effective interventions and measures that might reduce cognitive frailty caused by activity restrictions.

## Figures and Tables

**Table 1:** Baseline characteristics of the participants according to cognitive frailty status.

Characteristics	Total (*n* = 408)	Cognitive Frailty Status		*p*-Value
		CF (*n* = 164)	NCF (*n* = 244)	
Age (years), mean ± SD	70.54 ± 5.49	71.98 ± 6.46	69.58 ± 4.49	
Age group (years)				
65–69	222 (54.4)	68 (41.5)	154 (63.1)	< 0.001 **
70–79	146 (35.8)	69 (42.1)	77 (31.6)	
≥80	40 (9.8)	27 (16.5)	13 (5.3)	
Sex				
Male	167 (40.9)	61 (37.2)	106 (43.4)	0.219
Female	241 (59.1)	103 (62.8)	138 (56.6)	
Marital status				
Married	254 (62.3)	96 (58.5)	158 (64.8)	0.213
Single/divorced/widowed	154 (37.7)	68 (41.5)	86 (35.2)	
Educational level				
No education	10 (2.5)	4 (2.4)	6 (2.5)	< 0.001 **
Primary school (7 years)	359 (88.0)	157 (95.7)	202 (82.8)	
Secondary school and above (≥8 years)	39 (9.6)	3 (1.8)	36 (14.8)	
History of underlying diseases				
Hypertension	214 (52.5)	91 (55.5)	123 (50.4)	0.363
Diabetes mellitus	67 (16.4)	36 (22.0)	31 (12.7)	0.015 *
Hyperlipidemia	72 (17.6)	30 (18.3)	42 (17.2)	0.792
Osteoporosis/gout	19 (4.7)	9 (5.5)	10 (4.1)	0.633
Heart disease	17 (4.2)	9 (5.5)	8 (3.3)	0.316
Current drinking, *n* (%)	61 (15.0)	14 (8.5)	47 (19.3)	0.003 *
Current smoking, *n* (%)	25 (6.1)	8 (4.9)	17 (7.0)	0.564
BMI (kg/m^2^), mean ± SD	22.86 ± 3.87	22.73 ± 3.92	22.94 ± 3.84	
Underweight (<18.5 kg/m^2^) Normal weight (18.5–22.9 kg/m^2^) Overweight (23.0–24.9 kg/m^2^) Obese (>25.0 kg/m^2^)	42 (10.6) 174 (43.7) 81 (20.4) 101 (25.4)	20 (12.7) 66 (41.8) 30 (19.0) 42 (26.6)	22 (9.2) 108 (45.0) 51 (21.3) 59 (24.6)	0.636

Abbreviations: CF, cognitive frailty, NCF, non-cognitive frailty, SD, standard deviation; BMI, body mass index. Categorical variables are presented as a percentage, and continuous variables are presented as mean ± SD. The differences within baseline characteristics and cognitive frailty status were analyzed by chi-square test. * Significant association at *p* < 0.05; ** Significant association at *p* < 0.001.

**Table 2:** Comparison of the nutritional status, depression, sleep quality, and falling asleep at night between cognitively frail and non-cognitively frail groups.

	*n* (%)			
	Total (*n* = 408)	Cognitive Frailty Status		*p*-Value ^ab^
		CF (*n* = 164) ^a^	NCF (*n* = 244) ^b^	
MNA-SF score				
Total score, min.–max.	14, 2–14	14, 2–14	14, 5–14	
mean ± SD	10.29 ± 1.96	9.76 ± 2.25	10.65 ± 1.66	
median (IQR)	11.0 (3.0)	10.0 (4.0)	11.0 (2.0)	< 0.001 ^a,^ **
Nutritional status				
Normal	129 (31.6)	42 (25.6)	87 (35.7)	< 0.001 ^a,^ **
At risk of malnutrition	237 (58.1)	93 (56.7)	144 (59.0)	
Malnourished	42 (10.3)	29 (17.7)	13 (5.3)	
TGSD-15 score				
Total score, min.-max.	15, 0–15	15, 0–15	15, 0–10	
mean ±SD	2.34 ± 2.11	3.14 ± 2.54	1.80 ± 1.56	
median (IQR)	2.0 (2.0)	2.0 (3.0)	1.0 (2.0)	< 0.001 ^b,^ **
Depression				
No	359 (88.0)	126 (77.6)	233 (95.5)	< 0.001 ^a,^ **
Yes	49 (12.0)	38 (23.2)	11 (4.5)	
Global PSQI score				
Total score, min.-max.	21, 1–14	21, 3–14	21, 1–14	
mean ±SD	6.08 ± 2.47	6.49 ± 2.72	5.80 ± 2.26	
median (IQR)	5.0 (3.0)	6.0 (4.0)	5 (3.0)	0.016 ^b,^ *
Sleep quality				
Good	359 (88.0)	126 (77.6)	233 (95.5)	0.017 *
Bad	202 (49.5)	93 (56.7)	109 (44.7)	
Falling asleep at night (min)				
mean ± SD	25.56 ± 38.59	32.89 ± 51.35	20.64 ± 25.77	
median (IQR)	10.0 (25.0)	10.0 (25.0)	10.0 (25.0)	0.078 ^b^
Falling asleep at night				
Short period (≤30 min)	339 (83.1)	127 (77.4)	212 (86.9)	0.013 ^a,^ *
Long period (>30 min)	69 (16.9)	37 (22.6)	32 (13.1)	

Abbreviations: CF, cognitive frailty; NCF, non-cognitive frailty; min, minimum; max, maximum; SD, standard deviation; IQR, interquartile range. Statistical analysis was performed by ^a^ chi-square, ^b^ Mann–Whitney U test. * Significant association at *p* < 0.05; ** Significant association at *p* < 0.001.

**Table 3:** The associations between demographic and health characteristics, nutritional status, depression, sleep quality, falling asleep at night, and cognitive frailty.

Variables (*n* = 408)	Model A		Model B		Model C		Model D		Model E	
	COR, 95%CI	*p-*Value	AOR, 95%CI	*p-*Value	AOR, 95%CI	*p-*Value	AOR, 95%CI	*p-*Value	AOR, 95%CI	*p-*Value
Age (years)										
65–69	Ref.		Ref.		Ref.		Ref.		Ref.	
70–79	2.029, 1.317 to 3.127	0.001 **	2.017, 1.278 to 3.183	0.003 *	2.046, 1.283 to 3.263	0.003 *	2.186, 1.372 to 3.485	0.001 **	2.020, 1.277 to 3.194	0.003 *
>80 years	4.704, 2.288 to 9.669	<0.001 **	4.080, 1.919 to 8.672	<0.001 **	3.832, 1.771 to 8.293	0.001 **	4.019, 1.808 to 8.594	<0.001 **	3.954, 1.845 to 8.475	<0.001 **
Education level										
No education	8.000, 1.420 to 45.059	0.018 *	5.059, 0.802 to 31.909	0.085	4.047, 0.601 to 27.268	0.151	5.090, 0.792 to 32.700	0.086	5.018, 0.778 to 32.363	0.090
Primary school	9.327, 2.820 to 0.846	<0.001 **	7.596, 2.227 to 25.910	0.001 **	6.479, 1.919 to 21.876	0.003 *	7.460, 2.199 to 25.303	0.001 **	7.535, 2.211 to 25.682	0.001 **
Secondary school and above	Ref.		Ref.		Ref.		Ref.		Ref.	
Diabetes mellitus	1.932, 1.140 to 3.276	0.014 *	1.827, 1.044 to 3.196	0.035 *	NS		1.798, 1.025 to 3.153	0.041 *	1.826, 1.041 to 3.202	0.036 *
Nutritional status										
Normal	Ref.		Ref.		Ref.		Ref.		Ref.	
At risk of malnutrition	1.338, 0.852 to 2.101	0.206	1.327, 0.821 to 2.145	0.249	1.665, 0.818 to 2.180	0.248	1.311, 0.809 to 2.126	272	1.299, 0.801 to 2.106	0.289
Malnourished	4.621, 2.181 to 9.789	<0.001 **	3.786, 1.719 to 8.335	0.001 **	3.499, 1.547 to 7.914	0.003 *	3.498, 1.576 to 7.767	0.002 *	3.715, 1.675 to 8.237	0.001 **
Depression	6.388, 3.156 to 12.931	<0.001 **			5.003, 2.399 to 10.434	<0.001 **				
Sleep quality status	1.622, 1.089 to 2.417	0.017 *					1.613, 1.041 to 2.500	0.032 *		
Falling asleep at night	1.930, 1.145 to 3.252	0.014 *							1.809, 1.022 to 3.203	0.042 *

Abbreviations: OR, odds ratio; COR, crude odds ratio; AOR, adjusted odds ratio; CI, confidence interval; Ref., reference group; NS, non-significant. Model A, unadjusted; Model B, the association between nutritional status and cognitive frailty; Model C, the association between nutritional status, depression, and cognitive frailty; Model D, the association between nutritional status, sleep quality, and cognitive frailty; Model E, the association between nutritional status, falling asleep at night and cognitive frailty. Statistical analysis was performed by binary logistic regression and multivariate logistic regression with forward stepwise method. Adjusted for potential covariables of models B, C, D, and E were age, education level, diabetes mellitus, and current drinking. Reference group was non-cognitive frailty. * Significant association at *p* < 0.05; ** Significant association at *p* < 0.001.

## Data Availability

The data presented in this study are available on request from the corresponding author on reasonable request.

## Supplementary Materials

The following supporting information can be downloaded at: https://www.mdpi.com/article/10.3390/nu15132849/s1, Table S1: Descriptions of physical frailty parameters and cognitive components in cognitively frail and non-cognitively frail groups; Table S2: Comparison of nutritional status using MNA-SF screening in cognitively frail and non-cognitively frail groups; Table S3: Comparison of the percentage of TGDS-15 questions between cognitively frail and non-cognitively frail groups; Table S4: Descriptives of PSQI components in cognitively frail and non-cognitively frail groups; Table S5: Comparison of sleeping quality status and its components in cognitively frail and non-cognitively frail groups.

## References

[1] Lebrasseur A., Fortin-Bédard N., Lettre J., Raymond E., Bussières E.L., Lapierre N., Faieta J., Vincent C., Duchesne L., Ouellet M.C. (2021). Impact of the COVID-19 Pandemic on Older Adults: Rapid Review. JMIR Aging.

[2] Alhalaseh L., Kasasbeh F., Al-Bayati M., Haikal L., Obeidat K., Abuleil A., Wilkinson I. (2022). Loneliness and Depression among Community Older Adults during the COVID-19 Pandemic: A cross-sectional study. Psychogeriatrics.

[3] Chen P., Mao L., Nassis G.P., Harmer P., Ainsworth B.E., Li F. (2020). Coronavirus disease (COVID-19): The need to maintain regular physical activity while taking precautions. J. Sport. Health Sci..

[4] Palmer K., Monaco A., Kivipelto M., Onder G., Maggi S., Michel J.P., Prieto R., Sykara G., Donde S. (2020). The potential long-term impact of the COVID-19 outbreak on patients with non-communicable diseases in Europe: Consequences for healthy ageing. Aging Clin. Exp. Res..

[5] Di Santo S.G., Franchini F., Filiputti B., Martone A., Sannino S. (2020). The Effects of COVID-19 and Quarantine Measures on the Lifestyles and Mental Health of People over 60 at Increased Risk of Dementia. Front. Psychiatry.

[6] Furtado G.E., Caldo A., Rieping T., Filaire E., Hogervorst E., Teixeira A.M.B., Ferreira J.P. (2018). Physical frailty and cognitive status over-60 age populations: A systematic review with meta-analysis. Arch. Gerontol. Geriatr..

[7] Kelaiditi E., Cesari M., Canevelli M., van Kan G.A., Ousset P.J., Gillette-Guyonnet S., Ritz P., Duveau F., Soto M.E., Provencher V. (2013). Cognitive frailty: Rational and definition from an (I.A.N.A./I.A.G.G.) International Consensus Group. J. Nutr. Health Aging.

[8] Ruan Q., Yu Z., Chen M., Bao Z., Li J., He W. (2015). Cognitive frailty, a novel target for the prevention of elderly dependency. Ageing Res. Rev..

[9] Walston J., McBurnie M.A., Newman A., Tracy R.P., Kop W.J., Hirsch C.H., Gottdiener J., Fried L.P., Cardiovascular Health Study (2002). Frailty and activation of the inflammation and coagulation systems with and without clinical comorbidities: Results from the cardiovascular health study. Arch. Intern. Med..

[10] Sargent L., Nalls M., Starkweather A., Hobgood S., Thompson H., Amella E.J., Singleton A. (2018). Shared biological pathways for frailty and cognitive impairment: A systematic review. Ageing Res. Rev..

[11] Álvarez-Satta M., Berna-Erro A., Carrasco-Garcia E., Alberro A., Saenz-Antoñanzas A., Vergara I., Otaegui D., Matheu A. (2020). Relevance of oxidative stress and inflammation in frailty based on human studies and mouse models. Aging.

[12] Xue Q.L. (2011). The frailty syndrome: Definition and natural history. Clin. Geriatr. Med..

[13] Fried L.P., Tangen C.M., Walston J., Newman A.B., Hirsch C., Gottdiener J., Seeman T., Tracy R., Kop W.J., Burke G. (2001). Frailty in older adults: Evidence for a phenotype. J. Gerontol. A Biol. Sci. Med. Sci..

[14] Traykov L., Raoux N., Latour F., Gallo L., Hanon O., Baudic S., Bayle C., Wenisch E., Remy P., Rigaud A.S. (2007). Executive functions deficit in mild cognitive impairment. Cogn. Behav. Neurol..

[15] Liampas I., Folia V., Morfakidou R., Siokas V., Yannakoulia M., Sakka P., Scarmeas N., Hadjigeorgiou G., Dardiotis E., Kosmidis M.H. (2023). Language Differences among Individuals with Normal Cognition, Amnestic and Non-Amnestic MCI, and Alzheimer’s Disease. Arch. Clin. Neuropsychol..

[16] Saunders N.L., Summers M.J. (2010). Attention and working memory deficits in mild cognitive impairment. J. Clin. Exp. Neuropsychol..

[17] Shimada H., Makizako H., Lee S., Doi T., Lee S., Tsutsumimoto K., Harada K., Hotta R., Bae S., Nakakubo S. (2016). Impact of Cognitive Frailty on Daily Activities in Older Persons. J. Nutr. Health Aging.

[18] Feng L., Zin Nyunt M.S., Gao Q., Feng L., Yap K.B., Ng T.P. (2017). Cognitive Frailty and Adverse Health Outcomes: Findings from the Singapore Longitudinal Ageing Studies (SLAS). J. Am. Med. Dir. Assoc..

[19] Pinto T.C.C., Machado L., Bulgacov T.M., Rodrigues-Júnior A.L., Costa M.L.G., Ximenes R.C.C., Sougey E.B. (2019). Is the Montreal Cognitive Assessment (MoCA) screening superior to the Mini-Mental State Examination (MMSE) in the detection of mild cognitive impairment (MCI) and Alzheimer’s Disease (AD) in the elderly?. Int. Psychogeriatr..

[20] Zhang T., Ren Y., Shen P., Jiang S., Yang Y., Wang Y., Li Z., Yang Y. (2021). Prevalence and Associated Risk Factors of Cognitive Frailty: A Systematic Review and Meta-Analysis. Front. Aging Neurosci..

[21] Seesen M., Sirikul W., Ruangsuriya J., Griffiths J., Siviroj P. (2021). Cognitive Frailty in Thai Community-Dwelling Elderly: Prevalence and Its Association with Malnutrition. Nutrients..

[22] Chye L., Wei K., Nyunt M.S.Z., Gao Q., Wee S.L., Ng T.P. (2018). Strong Relationship between Malnutrition and Cognitive Frailty in the Singapore Longitudinal Ageing Studies (SLAS-1 and SLAS-2). J. Prev. Alzheimers Dis..

[23] Kwan R.Y.C., Leung A.Y.M., Yee A., Lau L.T., Xu X.Y., Dai D.L.K. (2019). Cognitive Frailty and Its Association with Nutrition and Depression in Community-Dwelling Older People. J. Nutr. Health Aging.

[24] Bu Z., Huang A., Xue M., Li Q., Bai Y., Xu G. (2021). Cognitive frailty as a predictor of adverse outcomes among older adults: A systematic review and meta-analysis. Brain Behav..

[25] World Health Organization Malnutrition. https://www.who.int/health-topics/malnutrition#tab=tab_1.

[26] O’Connor D., Molloy A.M., Laird E., Kenny R.A., O’Halloran A.M. (2023). Sustaining an ageing population: The role of micronutrients in frailty and cognitive impairment. Proc. Nutr. Soc..

[27] Mustafa Khalid N., Haron H., Shahar S., Fenech M. (2022). Current Evidence on the Association of Micronutrient Malnutrition with Mild Cognitive Impairment, Frailty, and Cognitive Frailty among Older Adults: A Scoping Review. Int. J. Environ. Res. Public Health.

[28] Sawchuk C. Depression (Major Depressive Disorder), Mayo Clinic. https://www.mayoclinic.org/diseases-conditions/depression/symptoms-causes/syc-20356007.

[29] Soysal P., Veronese N., Thompson T., Kahl K.G., Fernandes B.S., Prina A.M., Solmi M., Schofield P., Koyanagi A., Tseng P.T. (2017). Relationship between depression and frailty in older adults: A systematic review and meta-analysis. Ageing Res. Rev..

[30] Da Mata F.A.F., Miranda Forte Gomes M., Lício Ferreira Santos J., Aparecida de Oliveira Duarte Y., Gomes Pereira M. (2021). Depression and frailty in older adults: A population-based cohort study. PLoS ONE.

[31] Zhang F.F., Peng W., Sweeney J.A., Jia Z.Y., Gong Q.Y. (2018). Brain structure alterations in depression: Psychoradiological evidence. CNS Neurosci. Ther..

[32] Butters M.A., Young J.B., Lopez O., Aizenstein H.J., Mulsant B.H., Reynolds C.F., DeKosky S.T., Becker J.T. (2008). Pathways linking late-life depression to persistent cognitive impairment and dementia. Dialogues Clin. Neurosci..

[33] Diniz B.S., Butters M.A., Albert S.M., Dew M.A., Reynolds C.F. (2013). Late-life depression and risk of vascular dementia and Alzheimer’s disease: Systematic review and meta-analysis of community-based cohort studies. Br. J. Psychiatry..

[34] Wennberg A.M.V., Louis E.K.S. (2019). Interconnectedness among frailty, sleep, and cognition: Recent findings and clinical implications. Int. Psychogeriatr..

[35] Kaur S., Banerjee N., Miranda M., Slugh M., Sun-Suslow N., McInerney K.F., Sun X., Ramos A.R., Rundek T., Sacco R.L. (2019). Sleep quality mediates the relationship between frailty and cognitive dysfunction in non-demented middle aged to older adults. Int. Psychogeriatr..

[36] Liu S., Hu Z., Guo Y., Zhou F., Li S., Xu H. (2022). Association of sleep quality and nap duration with cognitive frailty among older adults living in nursing homes. Front. Public Health.

[37] Sun X.H., Ma T., Yao S., Chen Z.K., Xu W.D., Jiang X.Y., Wang X.F. (2020). Associations of sleep quality and sleep duration with frailty and pre-frailty in an elderly population Rugao longevity and ageing study. BMC Geriatr..

[38] Nelson K.L., Davis J.E., Corbett C.F. (2022). Sleep quality: An evolutionary concept analysis. Nurs. Forum.

[39] Fung C.H., Vitiello M.V., Alessi C.A., Kuchel G.A. (2016). Report and Research Agenda of the American Geriatrics Society and National Institute on Aging Bedside-to-Bench Conference on Sleep, Circadian Rhythms, and Aging: New Avenues for Improving Brain Health, Physical Health, and Functioning. J. Am. Geriatr. Soc..

[40] Zhao Y., Lu Y., Zhao W., Wang Y., Ge M., Zhou L., Yue J., Dong B., Hao Q. (2021). Long sleep duration is associated with cognitive frailty among older community-dwelling adults: Results from West China Health and Aging Trend study. BMC Geriatr..

[41] Franceschini C., Musetti A., Zenesini C., Palagini L., Scarpelli S., Quattropani M.C., Lenzo V., Freda M.F., Lemmo D., Vegni E. (2020). Poor Sleep Quality and Its Consequences on Mental Health during the COVID-19 Lockdown in Italy. Front. Psychol..

[42] Foundation of Thai Gerontology Research and Development Institute (TGRI) (2021). Situation of the Thai Older Persons 2020.

[43] World Health Organization WHO Coronavirus (COVID-19) Dashboard. Thailand Situation. https://covid19.who.int/region/searo/country/th.

[44] Peduzzi P., Concato J., Kemper E., Holford T.R., Feinstein A.R. (1996). A simulation study of the number of events per variable in logistic regression analysis. J. Clin. Epidemiol..

[45] Boongird P. Mental State Examination T10, Dementia Association of Thailand Newsletter. https://thaidementia.com/news/assets/files/DAT_news_letter_10.pdf.

[46] International Obesity Task Force (1999). Asia-Pacific Regional Obesity Guidelines.

[47] Mitnitski A.B., Mogilner A.J., Rockwood K. (2001). Accumulation of deficits as a proxy measure of aging. Sci. World J..

[48] Julayanont P., Tangwongchai S., Hemrungrojn S., Tunvirachaisakul C., Phanthumchinda K., Hongsawat J., Suwichanarakul P., Thanasirorat S., Nasreddine Z.S. (2015). The Montreal Cognitive Assessment-Basic: A Screening Tool for Mild Cognitive Impairment in Illiterate and Low-Educated Elderly Adults. J. Am. Geriatr. Soc..

[49] Nasreddine Z.S., Phillips N.A., Bédirian V., Charbonneau S., Whitehead V., Collin I., Cummings J.L., Chertkow H. (2005). The Montreal Cognitive Assessment, MoCA: A brief screening tool for mild cognitive impairment. J. Am. Geriatr. Soc..

[50] Lundin H., Sääf M., Strender L.E., Mollasaraie H.A., Salminen H. (2012). Mini nutritional assessment and 10-year mortality in free-living elderly women: A prospective cohort study with 10-year follow-up. Eur. J. Clin. Nutr..

[51] Kaiser M.J., Bauer J.M., Ramsch C., Uter W., Guigoz Y., Cederholm T., Thomas D.R., Anthony P., Charlton K.E., Maggio M. (2009). Validation of the Mini Nutritional Assessment short-form (MNA-SF): A practical tool for identification of nutritional status. J. Nutr. Health Aging.

[52] Lorenzo-López L., Maseda A., de Labra C., Regueiro-Folgueira L., Rodríguez-Villamil J.L., Millán-Calenti J.C. (2017). Nutritional determinants of frailty in older adults: A systematic review. BMC Geriatr..

[53] Cereda E., Pedrolli C., Klersy C., Bonardi C., Quarleri L., Cappello S., Turri A., Rondanelli M., Caccialanza R. (2016). Nutritional status in older persons according to healthcare setting: A systematic review and meta-analysis of prevalence data using MNA^®^. Clin. Nutr..

[54] Vellas B., Villars H., Abellan G., Soto M.E., Rolland Y., Guigoz Y., Morley J., Chumlea W.C., Salvá A., Rubenstein L.Z. (2006). Overview of the MNA—Its history and challenges. J. Nutr. Health Aging.

[55] Guigoz Y. (2006). The Mini Nutritional Assessment (MNA) review of the literature—What does it tell us?. J. Nutr. Health Aging.

[56] Sheikh J.I., Yesavage J.A. (1986). Geriatric Depression Scale (GDS): Recent findings and development of a short version. Clin. Gerontol..

[57] Yesavage J.A., Brink T.L., Rose T.L., Lum O., Huang V., Adey M., Leirer V.O. (1982). Development and validation of a geriatric depression screening scale: A preliminary report. J. Psychiatr. Res..

[58] Park S.H., Kwak M.J. (2021). Performance of the Geriatric Depression Scale-15 with Older Adults Aged over 65 Years: An Updated Review 2000–2019. Clin. Gerontol..

[59] Wongpakaran N., Wongpakaran T., Van Reekum R. (2013). The Use of GDS-15 in Detecting MDD: A Comparison between Residents in a Thai Long-Term Care Home and Geriatric Outpatients. J. Clin. Med. Res..

[60] Wongpakaran N., Wongpakaran T. (2012). Prevalence of major depressive disorders in long-term care facilities: A report northern Thailand. Psychogeriatrics.

[61] Buysse D.J., Reynolds C.F., Monk T.H., Berman S.R., Kupfer D.J. (1989). The Pittsburgh Sleep Quality Index: A new instrument for psychiatric practice and research. Psychiatry Res..

[62] Zhang C., Zhang H., Zhao M., Li Z., Cook C.E., Buysse D.J., Zhao Y., Yao Y. (2020). Reliability, Validity, and Factor Structure of Pittsburgh Sleep Quality Index in Community-Based Centenarians. Front. Psychiatry.

[63] Methipisit T., Mungthin M., Saengwanitch S., Ruangkana P., Chinwarun Y., Ruangkanchanasetr P., Panichkul S., Ukritchon S., Mahakit P., Sithinamsuwan P. (2016). The Development of Sleep Questionnaires Thai Version (ESS, SA-SDQ, and PSQI): Linguistic Validation, Reliability Analysis and Cut-Off Level to Determine Sleep Related Problems in Thai Population. J. Med. Assoc. Thai..

[64] Sitasuwan T., Bussaratid S., Ruttanaumpawan P., Chotinaiwattarakul W. (2014). Reliability and validity of the Thai version of the Pittsburgh Sleep Quality Index. J. Med. Assoc. Thai.

[65] Carpenter J.S., Andrykowski M.A. (1998). Psychometric evaluation of the Pittsburgh Sleep Quality Index. J. Psychosom. Res..

[66] Fang W., Li Z., Wu L., Cao Z., Liang Y., Yang H., Wang Y., Wu T. (2013). Longer habitual afternoon napping is associated with a higher risk for impaired fasting plasma glucose and diabetes mellitus in older adults: Results from the Dongfeng-Tongji cohort of retired workers. Sleep Med..

[67] von Elm E., Altman D.G., Egger M., Pocock S.J., Gøtzsche P.C., Vandenbroucke J.P. (2008). The Strengthening the Reporting of Observational Studies in Epidemiology (STROBE) statement: Guidelines for reporting observational studies. J. Clin. Epidemiol..

[68] Kim H., Awata S., Watanabe Y., Kojima N., Osuka Y., Motokawa K., Sakuma N., Inagaki H., Edahiro A., Hosoi E. (2019). Cognitive frailty in community-dwelling older Japanese people: Prevalence and its association with falls. Geriatr. Gerontol. Int..

[69] Brigola A.G., Alexandre T.D.S., Inouye K., Yassuda M.S., Pavarini S.C.I., Mioshi E. (2019). Limited formal education is strongly associated with lower cognitive status, functional disability and frailty status in older adults. Dement. Neuropsychol..

[70] Zahodne L.B., Stern Y., Manly J.J. (2015). Differing effects of education on cognitive decline in diverse elders with low versus high educational attainment. Neuropsychology.

[71] Hoogendijk E.O., van Hout H.P., Heymans M.W., van der Horst H.E., Frijters D.H., Broese van Groenou M.I., Deeg D.J., Huisman M. (2014). Explaining the association between educational level and frailty in older adults: Results from a 13-year longitudinal study in the Netherlands. Ann. Epidemiol..

[72] Hausdorff J.M., Schweiger A., Herman T., Yogev-Seligmann G., Giladi N. (2008). Dual-task decrements in gait: Contributing factors among healthy older adults. J. Gerontol. A Biol. Sci. Med. Sci..

[73] Won E., Choi S., Kang J., Kim A., Han K.M., Chang H.S., Tae W.S., Son K.R., Joe S.H., Lee M.S. (2016). Association between reduced white matter integrity in the corpus callosum and serotonin transporter gene DNA methylation in medication-naive patients with major depressive disorder. Transl. Psychiatry.

[74] Kerling A., Hartung D., Stubbs B., Kück M., Tegtbur U., Grams L., Weber-Spickschen T.S., Kahl K.G. (2018). Impact of aerobic exercise on muscle mass in patients with major depressive disorder: A randomized controlled trial. Neuropsychiatr. Dis. Treat..

[75] Wang L., Wang X., Song P., Han P., Fu L., Chen X., Yu H., Hou L., Yu X., Zhang Y. (2020). Combined Depression and Malnutrition As an Effective Predictor of First Fall Onset in a Chinese Community-Dwelling Population: A 2-Year Prospective Cohort Study. Rejuvenation Res..

[76] Szeto C.C., Chan G.C., Ng J.K., Chow K.M., Kwan B.C., Cheng P.M., Kwong V.W., Law M.C., Leung C.B., Li P.K. (2018). Depression and Physical Frailty Have Additive Effect on the Nutritional Status and Clinical Outcome of Chinese Peritoneal Dialysis. Kidney Blood Press. Res..

[77] Casagrande M., Forte G., Favieri F., Corbo I. (2022). Sleep Quality and Aging: A Systematic Review on Healthy Older People, Mild Cognitive Impairment and Alzheimer’s Disease. Int. J. Environ. Res. Public Health.

[78] Spira A.P., Gonzalez C.E., Venkatraman V.K., Wu M.N., Pacheco J., Simonsick E.M., Ferrucci L., Resnick S.M. (2016). Sleep Duration and Subsequent Cortical Thinning in Cognitively Normal Older Adults. Sleep.

[79] Pourmotabbed A., Boozari B., Babaei A., Asbaghi O., Campbell M.S., Mohammadi H., Hadi A., Moradi S. (2020). Sleep and frailty risk: A systematic review and meta-analysis. Sleep Breath..

[80] Mangiafico R.A., Sarnataro F., Mangiafico M., Fiore C.E. (2006). Impaired cognitive performance in asymptomatic peripheral arterial disease: Relation to C-reactive protein and D-dimer levels. Age Ageing.

[81] Thichumpa W., Howteerakul N., Suwannapong N., Tantrakul V. (2018). Sleep quality and associated factors among the elderly living in rural Chiang Rai, northern Thailand. Epidemiol. Health.

[82] Gupta R., Grover S., Basu A., Krishnan V., Tripathi A., Subramanyam A., Nischal A., Hussain A., Mehra A., Ambekar A. (2020). Changes in sleep pattern and sleep quality during COVID-19 lockdown. Indian J. Psychiatry.

[83] Borrescio-Higa F., Droller F., Valenzuela P. (2022). Financial Distress and Psychological Well-Being during the COVID-19 Pandemic. Int. J. Public Health.

[84] Burton E., Lewin G., Boldy D. (2015). Physical activity preferences of older home care clients. Int. J. Older People Nurs..

[85] Kitamura K., Watanabe Y., Nakamura K., Takano C., Hayashi N., Sato H., Someya T. (2021). Short daytime napping reduces the risk of cognitive decline in community-dwelling older adults: A 5-year longitudinal study. BMC Geriatr..

[86] Abdelhafiz A.H., Sinclair A.J. (2019). Cognitive Frailty in Older People with Type 2 Diabetes Mellitus: The Central Role of Hypoglycaemia and the Need for Prevention. Curr. Diab Rep..

[87] Khandelwal D., Dutta D., Chittawar S., Kalra S. (2017). Sleep Disorders in Type 2 Diabetes. Indian J. Endocrinol. Metab..

[88] Fu Z., Wang F., Dang X., Zhou T. (2022). The association between diabetes and nocturia: A systematic review and meta-analysis. Front. Public Health.

