# Supplementary material for: Malnutrition, Depression, Poor Sleep Quality, and Difficulty Falling Asleep at Night Are Associated with a Higher Risk of Cognitive Frailty in Older Adults during the COVID-19 Restrictions
Source: Nutrients. 2023 Jun 23;15(13):2849. doi: 10.3390/nu15132849 (PMC10343894; doi:10.3390/nu15132849)
Supplement: Supplementary file 1 [file nutrients-15-02849-s001.zip › nutrients-2471291-supplementary.pdf]

**Supplementary Table S1**. Descriptions of physical frailty parameters and cognitive components in cognitively frail and non-cognitively frail groups.

| | Parameters/Components | *n* (%), 95%CI | | | *P*–value |
|---|---|---|---|---|---|
| | | Total (*n* = 408) | Cognitive frailty status | | |
| | | | CF (n = 164) | NCF (n = 244) | |
| Physical frailty | Self-reported exhaustion | 170 (41.7), 37.0 to 46.0 | 113 (68.9), 62.0 to 76.0 | 57 (23.4), 18.0 to 29.0 | < 0.001** |
| | Unintended weight loss | 122 (29.9), 25.4 to 34.4 | 86 (52.4), 44.7 to 60.1 | 36 (14.8), 10.3 to 19.4 | < 0.001** |
| | Weakness | 253 (62.0), 57.3 to 66.7 | 140 (85.4), 79.9 to 90.8 | 113 (46.3), 40.0 to 52.6 | < 0.001** |
| | Slow walking speed | 268 (65.7), 61.1 to 70.3 | 148 (90.2), 85.6 to 94.98 | 120 (49.2), 42.9 to 55.5 | < 0.001** |
| | Low physical activity | 84 (20.6), 16.6 to 24.5 | 60 (36.6), 29.1 to 44.0 | 24 (9.8), 6.1 to 13.6 | < 0.001** |
| | Physical frailty | | | | |
| | Non-frail | (58.6), 53.8 to 63.4 | | | |
| | Pre-frail/frail | (41.4), 36.6 to 46.22 | | | |
| Cognitive function impairment | Executive function | 363 (89.0), 85.9 to 92.0 | 159 (97.0), 94.3 to 99.6 | 204 (83.6), 78.9 to 88.3 | < 0.001 ** |
| | Fluency | 76 (18.6), 14.8 to 22.4 | 39 (23.8), 17.2 to 30.4 | 37 (15.2), 10.6 to 19.7 | < 0.001** |
| | Orientation | 22 (5.4), 3.2 to 7.6 | 5 (3.0), 0.4 to 5.7 | 17 (7.0), 3.8 to 10.2 | 0.039* |
| | Calculation | 25 (6.1), 3.8 to 8.5 | 15 (9.1), 4.7 to 13.6 | 10 (4.1), 1.6 to 6.6 | 0.004* |
| | Abstraction | 86 (21.1), 17.1 to 25.1 | 49 (29.9), 22.8 to 37.0 | 37 (15.2), 10.6 to 19.7 | < 0.001** |
| | Delayed recall | 147 (36.0), 31.4 to 40.7 | 69 (42.1), 34.4 to 49.7 | 78 (32.0), 26.1 to 37.9 | 0.052 |
| | Visuoperception | 76 (18.6), 14.8 to 22.1 | 43 (26.2), 19.4 to 33.0 | 33 (13.5), 9.2 to 17.8 | 0.643 |
| | Naming | 1 (0.2), 0.2 to 0.7 | 0 (0.0) | 1 (0.4) 0.0 to 1.2 | 0.001** |
| | Attention | 140 (34.3), 29.7 to 38.9 | 75 (45.7), 38.0 to 53.4 | 65 (26.6), 21.1 to 32.2 | < 0.001** |
| | Alternative attention | 147 (36.0), 21.3 to 40.7 | 79 (48.2), 40.4 to 55.9 | 68 (27.9), 22.2 to 33.5 | < 0.001** |
| | Mild cognitive impairment | | | | |
| | No | 63 (15.4), 11.9 to 19.0 | | | |
| | Yes | 345 (84.6), 81.0 to 88.1 | | | |

Abbreviations: CF, Cognitive frailty; NCF, non-cognitive frailty; CI, confidence interval. * Significant association at $p < 0.05$; ** Significant association at $p < 0.001$.

**Supplementary Table S2.** Comparison of nutritional status using MNA-SF screening in cognitively frail and non-cognitively frail groups.

| MNA-SF Questions | Total (N= 408) | Cognitive frailty status | | *P*-value |
|---|---|---|---|---|
| | | CF (*n* = 164) | NCF (*n* = 244) | |
| Has food intake declined over the past 3 months due to loss of appetite, digestive problems, chewing or swallowing difficulties? | | | | |
| Severe decrease in food intake | 31 (7.6) | 19 (11.6) | 12 (4.9) | < 0.001**, a |
| Moderate decrease in food intake | 104 (25.5) | 62 (37.8) | 42 (17.2) | |
| No decrease in food intake | 273 (66.9) | 83 (50.6) | 190 (77.9) | |
| Weight loss during the last 3 months | | | | |
| Weight loss greater than 3 kg (6.6 lbs) | 31 (7.6) | 19 (11.6) | 12 (4.9) | < 0.001**, a |
| Does not know | 13 (3.2) | 9 (5.5) | 4 (1.6) | |
| Weight loss between 1 and 3 kg (2.2 and 6.6 lbs) | 93 (22.8) | 49 (29.9) | 44 (18.0) | |
| No weight loss | 271 (66.4) | 87 (32.1) | 184 (75.4) | |
| Mobility | | | | |
| Bed or chair bound | 1 (0.2) | 0 (0.0) | 1 (0.4) | 0.161 b |
| Able to get out of bed/ chair but does not go out | 2 (0.5) | 2 (1.2) | 0 (0.0) | |
| Goes out | 405 (99.3) | 162 (98.8) | 243 (99.6) | |
| Has suffered psychological stress or acute disease in the past 3 months? | | | | |
| Yes | 42 (10.3) | 25 (15.2) | 17 (7.0) | 0.008*, a |
| No | 366 (89.7) | 139 (84.8) | 227 (93.0) | |
| Neuropsychological problems | | | | |
| Severe dementia or depression | 238 (58.3) | 86 (52.4) | 152 (62.3) | 0.083 b |
| Mild dementia | 162 (39.7) | 73 (44.5) | 89 (36.5) | |
| No psychological problems | 8 (2.0) | 5 (3.0) | 3 (1.2) | |
| Body Mass Index (BMI) ($kg/m^2$) | | | | |
| Less than 19 | 48 (11.9) | 21 (13.3) | 27 (11.1) | 0.785 a |
| 19 to less than 21 | 79 (19.7) | 31 (19.6) | 48 (19.7) | |
| 21 to less than 23 | 88 (21.9) | 31 (19.6) | 57 (23.4) | |
| 23 or greater | 187 (46.5) | 75 (47.5) | 112 (45.9) | |

Abbreviations: CF, Cognitive frailty; NCF, non-cognitive frailty; MNA-SF, Mini Nutritional Assessment (Short form); Statistical analysis was performed by [a] Chi-square test and [b] Fisher's Exact test. * Significant association at $p < 0.05$; ** Significant association at $p < 0.001$.

**Supplementary Table S3**. Comparison of the percentage of TGDS-15 questions between cognitively frail and non-cognitively frail groups.

| TGDS-15 Questions | Response answer ≠ | Total (N= 408) | Cognitive frailty status | | *P*-value |
|---|---|---|---|---|---|
| | | | **CF (*n* = 164)** | **NCF (*n* = 244)** | |
| 1. Are you basically satisfied with your life? | **No** | 15 (3.7) | 5 (3.0) | 10 (4.1) | 0.581 [a] |
| 2. Have you dropped many of your activities or interests? | **Yes** | 112 (27.5) | 60 (36.6) | 52 (21.3) | 0.001**, [a] |
| 3. Do you feel that your life is empty? | **Yes** | 58 (14.2) | 37 (22.6) | 21 (8.6) | < 0.001**, [a] |
| 4. Do you often feel bored? | **Yes** | 68 (16.7) | 48 (29.3) | 20 (8.2) | < 0.001**, [a] |
| 5. Are you in good spirits most of the time? | **No** | 12 (2.9) | 8 (4.9) | 4 (1.6) | 0.074 [b] |
| 6. Are you afraid that something bad is going to happen to you? | **Yes** | 87 (21.3) | 46 (28.0) | 41 (16.8) | 0.007*, [a] |
| 7. Do you feel happy most of the time? | **No** | 9 (2.2) | 5 (3.0) | 4 (1.6) | 0.494 [b] |
| 8. Do you often feel helpless? | **Yes** | 31 (7.6) | 21 (12.8) | 10 (4.1) | 0.001**. [a] |
| 9. Do you prefer to stay at home, rather than going out and doing new things? | **Yes** | 265 (65.0) | 111 (67.7) | 154 (63.1) | 0.343 [a] |
| 10. Do you feel you have more problems with your memory than most? | **Yes** | 122 (29.9) | 62 (37.8) | 60 (24.6) | 0.004*, [a] |
| 11. Do you think it is wonderful to be alive? | **No** | 33 (8.1) | 21 (12.8) | 12 (4.9) | 0.004**, [a] |
| 12. Do you feel pretty worthless the way you are now? | **Yes** | 17 (4.2) | 13 (7.9) | 4 (1.6) | 0.002*, [a] |
| 13. Do you feel full of energy? | **No** | 46 (11.3) | 25 (15.2) | 21 (8.6) | 0.038*, [a] |
| 14. Do you feel that your situation is hopeless? | **Yes** | 25 (6.1) | 19 (11.6) | 6 (1.5) | < 0.001**, [a] |
| 15. Do you think that most people are better off than you are? | **Yes** | 52 (12.7) | 34 (20.7) | 18 (7.4) | < 0.001**, [a] |

≠ problems (response answers in bold) indicate depression; Statistical analysis was performed by [a] Chi-square test and [b] Fisher's Exact test; Abbreviations: CF, Cognitive frailty; NCF, non-cognitive frailty; TGDS, Thai Geriatric Depression Scores. * Significant association at $p < 0.05$; ** Significant association at $p < 0.001$.

**Supplementary Table S4.** Descriptives of PSQI components in cognitively frail and non-cognitively frail groups.

| PSQI components, n (%) | Total (*n* = 408) | Cognitive frailty CF (*n* = 164) | NCF (*n* = 244) | *P*–value |
|---|---|---|---|---|
| 1. Subjective sleep quality | | | | |
| Very good (0 score) | 336 (82.4) | 126 (76.8) | 210 (86.1) | 0.043 [*, a] |
| Fairly good (1 score) | 50 (12.3) | 24 (14.6) | 26 (10.7) | |
| Fairly bad (2 scores) | 18 (4.4) | 12 (7.3) | 6 (2.5) | |
| Very bad (3 scores) | 4 (1.0) | 2 (1.2) | 2 (0.8) | |
| 2. Sleep latency | | | | |
| 0 (score) | 188 (46.1) | 65 (39.6) | 123 (50.4) | 0.042 [*, b] |
| 1 – 2 (1 score) | 114 (27.9) | 46 (28.0) | 68 (27.9) | |
| 3 – 4 (2 scores) | 64 (15.7) | 29 (17.7) | 35 (14.3) | |
| 5 – 6 (scores) | 42 (10.3) | 24 (14.6) | 18 (7.4) | |
| 3. Sleep duration | | | | |
| > 7 (0 score) | 266 (65.2) | 107 (65.2) | 159 (65.2) | 0.177 [b] |
| 6 – 7 (1 score) | 59 (14.5) | 25 (15.2) | 34 (13.9) | |
| 5 – 6 (2 scores) | 67 (16.4) | 22 (13.4) | 45 (18.4) | |
| < 5 (3 scores) | 16 (3.9) | 10 (6.1) | 6 (2.5) | |
| 4. Sleep efficiency (%) | | | | |
| > 85 (0 score) | 335 (82.1) | 128 (78.0) | 207 (84.8) | 0.058 [b] |
| 75 – 84 (1 score) | 26 (6.4) | 11 (6.7) | 15 (6.1) | |
| 65 – 74 (2 scores) | 19 (4.7) | 7 (4.3) | 12 (4.9) | |
| < 65 (3 scores) | 28 (6.9) | 18 (11.0) | 10 (4.1) | |
| 5. Sleep disturbance (scores) | | | | |
| 0 (0 score) | 12 (2.9) | 7 (4.3) | 5 (2.0) | 0.202 [a] |
| 1 – 9 (1 score) | 300 (73.5) | 112 (68.3) | 188 (77.0) | |
| 10 – 18 (2 scores) | 90 (22.1) | 42 (25.6) | 48 (19.7) | |
| 19 – 27 (3 scores) | 6 (1.5) | 3 (1.8) | 3 (1.2) | |
| 6. Use of sleep medication | | | | |
| Not during past month (0 score) | 17 (4.2) | 7 (4.3) | 10 (4.1) | 0.637 [b] |
| Less than once a week (1 score) | 18 (4.4) | 8 (4.9) | 10 (4.1) | |
| Once or twice a week (2 scores) | 119 (29.2) | 53 (32.3) | 66 (27.0) | |
| Three or more than times a week (3 scores) | 254 (62.3) | 96 (23.5) | 158 (64.8) | |
| 7. Daytime dysfunction (scores) | | | | |
| 0 (0 score) | 286 (70.1) | 102 (62.2) | 184 (75.4) | 0.024 [*, a] |
| 1 – 2 (1 score) | 99 (24.3) | 49 (29.9) | 50 (20.5) | |
| 3 – 4 (2 scores) | 19 (4.7) | 11 (6.7) | 8 (3.3) | |
| 5 – 6 (3 scores) | 4 (1.0) | 2 (1.2) | 2 (0.8) | |

Abbreviations: CF, Cognitive frailty; NCF, non-cognitive frailty; PSQI, Pittsburgh Sleep Quality Index; Statistical analysis was performed by [a] Fisher's Exact test and [b] Chi-square test. * Significant association at $p < 0.05$.

**Supplementary Table S5.** Comparison of sleeping quality scores and its components in cognitively frail and non-cognitively frail groups.

| Sleep Quality | Mean SD, median [IQR] | | | *P*-value |
|---|---|---|---|---|
| | **Total** | **Cognitive frailty status** | | |
| | **(*n* = 408)** | **CF (*n* = 164)** | **NCF (*n* = 244)** | |
| Subjective sleep quality (scores) | 0.0 [0.0] | 0.0 [0.0] | 0.0 [0.0] | 0.013 * |
| Sleep latency (scores) | 1.0 [2.0] | 1.0 [2.0] | 0.0 [1.0] | 0.008 * |
| Sleep duration (scores) | 0.0 [1.0] | 0.0 [1.0] | 0.0 [0.0] | 0.960 |
| Sleep efficiency (scores) | 0.0 [0.0] | 0.0 [0.0] | 0.0 [0.0] | 0.055 * |
| Sleep disturbance (scores) | 1.0 [0.0] | 1.0 [1.0] | 1.0 [0.0] | 0.296 |
| Use of sleep medication (scores) | 3.0 [1.0] | 3.0 [1.0] | 3.0 [1.0] | 0.229 |
| Daytime dysfunction (scores) | 0.0 [1.0] | 0.0 [1.0] | 0.0 [0.0] | 0.004 * |

Abbreviations: CF, Cognitive frailty; NCF, non-cognitive frailty; SD, Standard deviation; IDR, Interquartile range; Statistical analysis was performed by the Mann-Whitney U test. * Significant association at $p < 0.05$.
